# The Effect of Autologous Platelet Lysate Eye Drops: An In Vivo Confocal Microscopy Study

**DOI:** 10.1155/2016/8406832

**Published:** 2016-04-20

**Authors:** Antonio M. Fea, Vittoria Aragno, Valeria Testa, Federica Machetta, Simone Parisi, Sergio D'Antico, Roberta Spinetta, Enrico Fusaro, Federico M. Grignolo

**Affiliations:** ^1^Department of Clinical Sciences, Ophthalmology Institute, University of Turin, 10122 Turin, Italy; ^2^Rheumatology Department, AOU Città della Salute e della Scienza di Torino, 10134 Turin, Italy; ^3^Blood Bank, AOU Città della Salute e della Scienza di Torino, 10134 Turin, Italy

## Abstract

*Purpose.* To determine the effectiveness of autologous platelet lysate (APL) eye drops in patients with primary Sjögren syndrome (SS) dry eye, refractory to standard therapy, in comparison with patients treated with artificial tears. We focused on the effect of APL on cornea morphology with the in vivo confocal microscopy (IVCM).* Methods.* Patients were assigned to two groups: group A used autologous platelet lysate QID, and group B used preservative-free artificial tears QID, for 90 days. Ophthalmological assessments included ocular surface disease index (OSDI), best corrected visual acuity (BCVA), Schirmer test, fluorescein score, and breakup time (BUT). A subgroup of patients in group A underwent IVCM: corneal basal epithelium, subbasal nerves, Langerhans cells, anterior stroma activated keratocytes, and reflectivity were evaluated.* Results.* 60 eyes of 30 patients were enrolled; in group A (*n* = 20 patients) mean OSDI, fluorescein score, and BUT showed significant improvement compared with group B (*n* = 10 patients). The IVCM showed a significant increase in basal epithelium cells density and subbasal nerve plexus density and number and a decrease in Langerhans cells density (*p* < 0.05).* Conclusion.* APL was found effective in the treatment of SS dry eye. IVCM seems to be a useful tool to visualize cornea morphologic modifications.

## 1. Introduction

Sjögren syndrome (SS) is a chronic multisystem autoimmune disease characterized by hypofunction of salivary and lacrimal glands [1]. The pathogenesis of the dysfunction is due to a T-lymphocyte mediated destruction of the exocrine glands [2].

The result of the immune-mediate infiltration of the lacrimal gland is the development of a severe dry eye syndrome (DES).

The mainstay of conventional therapy for dry eye is the application of preservative-free artificial eye drops, which provide lubrication of the surface of the eye. Based on the concept that inflammation has a key role in the pathogenesis of dry eye, different treatment options, such as corticosteroids and cyclosporine, are used as a second-line treatment in more severe dry eye [3, 4].

However, none of the commercially available artificial tear preparations and anti-inflammatory topical treatment have the properties of the human tears. They do not contain growth factors (GFs), such as transforming growth factor *β* (TGF-*β*), and other components, including vitamin A, fibronectin, and other cytokines, which are necessary for the maintenance of normal corneal epithelium [5]. In particular, GFs stimulate tissue healing by inducing mesenchymal and epithelial cells migration and proliferation in case of ocular surface damage [6].

Since some of these components are found in serum, and its composition is very similar to natural tears, the autologous serum (AS) has been used since 1984 [7] as a second-line therapy for dry eye. Nevertheless, its potential benefits have been questioned by a recent meta-analysis [8].

Platelet alfa granules are a major source of GFs and are rich in platelet derived growth factor (PDGF), which plays an important role in the maintenance of ocular surface and tear film stability. PDGF promotes the chemotaxis of fibroblasts, monocytes, and macrophages and stimulates the expression of TGF-*β* that inhibits metalloproteases and decreases inflammation [9]. These findings prompted the use of platelet rich plasma (PRP), platelet rich plasma in growth factors (PRGF), and autologous plasma rich in PDGFs eye drops (PRGD); indeed, recent studies on PRP [10], PRGF [11], and PRGD [12] have reported an improvement in both the objective and subjective outcomes in DES patients.

The use of in vivo confocal microscopy (IVCM) offers a completely new approach in the study of the ocular surface, with a noninvasive high resolution analysis [13, 14] that allows both a quantitative histopathological assessment of cornea damage and a qualitative evaluation of cellular and nerve properties [15–17].

IVCM has been used to analyze the morphology of cornea in DES and to study its relationship with the clinical evaluation. The morphological abnormalities that appear in patients with SS were first demonstrated in 2003 by Tuominen et al. [18] and then confirmed by other authors [19, 20]. A patchy corneal epithelium, an activation of anterior keratocytes, and an abnormal subbasal nerve plexus have been described. Some studies reported that these abnormalities are reversible by a topical treatment with hemocomponents that are able to restore epithelial integrity [21, 22].

In this study, we evaluated the efficacy of autologous platelet lysate (APL) eye drops in patients with primary SS refractory dry eye in comparison to artificial free preservatives tears. We focused on the histological effect that APL could have on corneal morphological modifications with a layer-by- layer analysis of the corneal ultrastructure in a sample of patients treated with APL.

## 2. Methods

This prospective case-control study was conducted from July 2014 to May 2015 at the University Eye Clinic of Turin. The study was conducted in accordance with the Declaration of Helsinki (1964) and approved by our Ethics Committee.

### 2.1. Patients Selection

We included patients with a diagnosis of SS according to the classification criteria of the American-European Consensus [1], a dry eye severity level ≥2 (Dry Eye Severity Grading Scheme, Workshop 2007), an ocular surface disease index (OSDI) ≥23, and a corneal fluorescein staining score ≥1 on Oxford scale. All patients were refractory for more than 2 months to previous conventional therapy (artificial tears, steroids, cyclosporine A, or autologous serum).

We excluded patients with ocular infections, previous corneal surgery (refractive surgery or corneal transplantation), positive tests for HBV, HCV, HIV, and fever, or sepsis. Platelet count had to be higher than 100 × 10^3^/*μ*L and Hb >10 g/dL.

Patients were enrolled and assigned to two groups, according to the randomization criteria 2 : 1. Group A patients were treated with eye drops and group B with preservative-free artificial tears (0.2% hyaluronic acid and TS-polysaccharide 0.2%). Both groups were treated for 90 days, 1 drop 4 QID.

Both eyes were examined in all subjects, and, for statistical analysis, both eyes were selected if responding to inclusion criteria and separately analyzed.

### 2.2. Autologous Platelet Lysate Eye Drops Preparation

The withdrawal and the APL eye drops preparation were conducted at the Blood Bank, AOU Città della Salute e della Scienza di Torino. A volume of 250 to 350 mL of venous peripheral blood was sampled from each patient, depending on platelet count, Hb value, total blood volume, and clinical condition; the autologous packed red blood cells separated from whole blood could be reinfused in patients having mild anemia (Hb < 11 g/dL). CompoFlow® T & B Fresenius Kabi bags, containing CPD anticoagulant, were used for the collection of blood. The blood was left for 60 min at room temperature and then centrifuged at 1580 rpm × 8 minutes (centrifuge Cryofuge 6000i) to obtain the PRP (platelet rich plasma) that underwent a second centrifugation (4000 rpm × 8 min). The PPP (platelet poor plasma) supernatant was partially removed to obtain a platelet concentration of 1.000 × 10^3^/*µ*L ± 10%. PRP was left at room temperature for 30 minutes, and then it was diluted by addition of 0.9% NaCl saline solution until a final concentration of 50% and platelets were lysed by three consecutive thermal shocks. Platelet lysate (PL) was centrifuged (4000 rpm × 8 min) and the supernatant PL was poured into a collection bag. The bag was connected to a COL.C30 (Biomed Device) kit and then aliquoted into 30 sterile vials (following Biomed Device instructions) and kept frozen at −80°C. A sample of the final preparation was used for bacterial culture (BacT/ALERT®) that had to be negative before the delivery to the patient.

Patients were instructed to keep APL frozen, to thaw the single daily dose the night before, and to store it at 4°C during the day. Thirty single doses of platelet lysate were delivered to group A subjects every 30 days. The schedule of use was two drops QID for 90 days.

### 2.3. Ophthalmological Assessment

Before and after 90 days of therapy, patients underwent an ophthalmologic assessment comprehensive of subjective symptoms evaluation by ocular surface disease index (OSDI), Schirmer test type I, breakup time (BUT), and corneal fluorescein staining (Oxford scale). Moreover, best corrected visual acuity (BCVA) was evaluated in Snellen lines and eyelid malposition or squamous metaplasia as well as anterior and posterior blepharitis (Efron Scale) [23], conjunctival folds (LIPCOF) [24], and Ocular Protection Index (OPI) were checked. Patients were then classified according to the Dry Eye Severity Grading Scheme and only those with grade ≥2 were enrolled in the study.

Patient's examination was always conducted by the same observer (AV).

In case of suspected conjunctivitis, the treatment was suspended and a microbial culture was performed to prescribe an appropriate local therapy, and patients took on again their original treatment after resolution.

### 2.4. In Vivo Corneal Confocal Microscopy Assessment

In vivo confocal microscopy was performed before and after 90 days of therapy with APL, on a sample of patients (subgroup 2) randomized from subjects treated with APL (group A), with Heidelberg Retina Tomograph (HRT II) in combination with Rostock Cornea Module (RCM, 63x/0.9 W. 670 nm, ∞/0; Zeiss, Jena, Germany) and CCD camera.

The microscopy was performed on both eyes of each patient after a topical instillation of anesthetic (0.4% oxybuprocaine hydrochloride). A drop of an ophthalmic gel (2.0 g hydroxypropyl methylcellulose) was applied on the ocular surface, as coupling medium, to improve the adhesion of the contact cap objective to the cornea. Proper alignment and positioning of the head were maintained with the help of a dedicated target mobile red fixation light for the contralateral eye.

After the exam, the integrity of the corneal surface was controlled at the slit lamp and patients were medicated with antibiotic eye drops (tobramycin ophthalmic solution 0.3%).

We examined the central corneal areas, where images of intermediate and deep epithelial layers and anterior stroma were acquired and analyzed.

Patient's examination was always conducted by the same observer (TV).

All images were reviewed, and three well-focused micrographs for each corneal scan were manually selected. The micrographs were assessed in a masked way by one observer; the mean value of series of three readings was recorded for each parameter.

**Figure 1:**
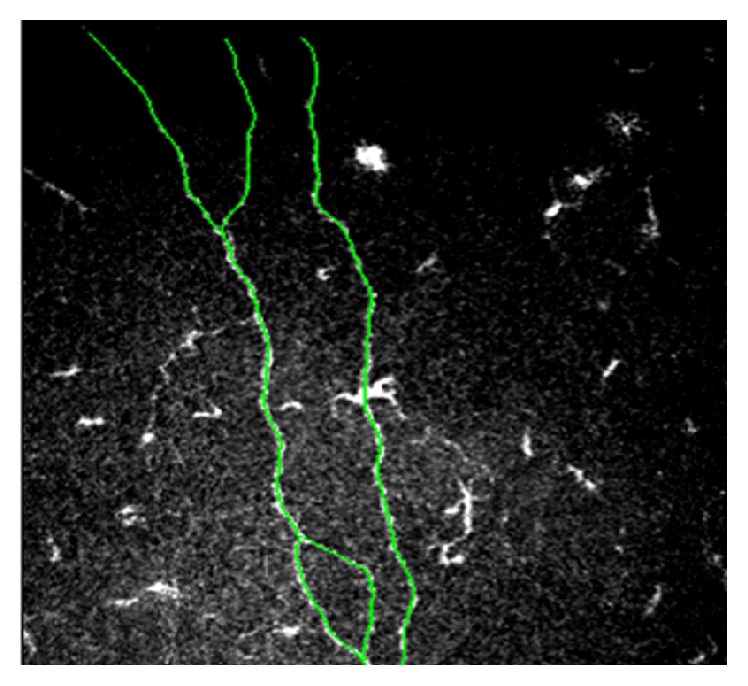
An example of neuron tracing with NeuronJ software. The plugin facilitates the tracing and quantification of nerves.

Evaluated parameters included the following:

- Basal epithelium cell density (cells/mm^2^): basal epithelial layer was considered 10 *µ*m above the membrane of Bowman. The counting was carried out within a region of interest of standardized size (region of interest [ROI] = 100 × 100 *µ*m) using the manual cell counting system offered with the software. The system automatically calculated the density by the function “cell count.”
- Number of subbasal nerve fibers (between Bowman's membrane and the basal layer of epithelial, where nerve fibers run parallel to the corneal surface): it is defined as the sum of nerve branches in each frame; images with the greatest number of visible nerve fibers were chosen for each series of acquisitions. Only fibers longer than 50 *µ*m were considered and counted as a separated branch.
- Nerve fiber density (*µ*m/160000 *μ*m^2^): it is defined as the sum of nerve fibers length (>50 *µ*m), considered in the area of 400 × 400 *μ*m (0.1589 mm^2^, defined as the widest area possible); it was performed using the function MEASUREMENTS > J GROUP MEASUREMENTS of NeuronJ, plugin software ImageJ (available at http://rsb.info.nih.gov/ij/) (Figure 1).
- Activated keratocytes density of the anterior stroma (number of activated keratocytes/mm^2^): number of activated keratocytes in the stroma of the Central Front is defined as hyperreflective and with cytoplasm extensions, 20 *µ*m under the Bowman layer. The counting was carried out using the manual cell counting system offered with the software in the region of interest (ROI) of 400 × 400 *μ*m (0.1589 mm^2^, defined as the widest area possible).
- Anterior stroma mean gray value: it is expressed with a value referred to as gray scale from 0 (black) to 255 (white) in Optical Units (OU), in relation to the anterior stroma reflectivity, with ImageJ software [25].
- Langerhans cells (LCs) density (LCs/mm^2^): dendritic cells number was manually counted in each corneal section at the level of subbasal nerves, selecting the images in which there was a greater density of LCs. The counting was carried out using the manual cell counting system offered with the software in the region of interest (ROI) of 400 × 400 *μ*m (0.1589 mm^2^, defined as the widest area possible).

### 2.5. Statistical Analysis

Descriptive statistics were produced for demographic, clinical, and laboratory variables. Mean and standard deviation were calculated for quantitative variables normally distributed; median and IQR were presented for not normally distributed quantitative variables and ordinal variables. Categorical variables were presented with frequencies and percentages.

A sample size of 50 eyes was calculated to detect a difference in OSDI between interventions of 50%, defining significance level of 0.05 and power of 70%.

The Wilcoxon test (*k* = 2) was used for quantitative continuous variables and for ordinal variables in order to assess statistically significant changes from the baseline. The nonparametric Mann-Whitney *U* test was chosen to compare the continuous quantitative variables and ordinal variables between the two groups; Fisher's exact test was used to compare the nominal categorical variables where appropriate. Performed tests were bilateral and the level of significance was set at 5%. The data were analyzed using the statistical software R.

## 3. Results

From July 2014 to May 2015, we enrolled 30 patients (mean age 59.5 ± 12.2 years) with primary Sjögren syndrome (selected from a starting group of 164 patients with primary SS followed up by the Rheumatology Department, the AOU Città della Salute e della Scienza di Torino, Turin). Group A consisted of 20 patients (40 eyes) and group B of 10 patients (20 eyes); demographic characteristics are summarized in Table 1. Dry eye duration was considered from the diagnosis of SS. The groups at baseline did not have significant differences (*p* > 0.05).

### 3.1. Ophthalmological Assessment

The ophthalmological parameters assessed are reported in Table 2.

None of the subjects presented eyelid abnormalities and squamous metaplasia.

**Figure 2:**
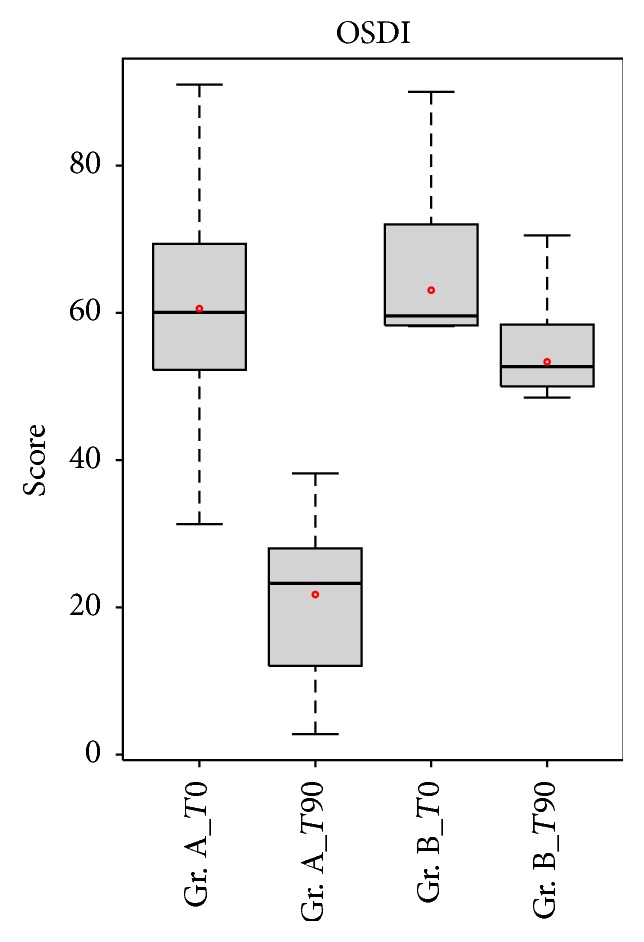
Comparison of OSDI score between patients in group A (APL) and patients in group B (artificial tears). OSDI score was significantly lower in group A patients compared with the group B patients after treatment (*p* < 0.05).

Mean OSDI score statistically decreased in group A from 60, 56 ± 17, and 69 to 21, 75 ± 9, and 86 after 90 days with APL therapy (Δ = 38, 82, *p* value = 0.00438). In group B, it slightly decreased from 60, 67 ± 16, and 19 to 53, 60 ± 14, and 91 (Δ = 7, 07). The differences between the two groups after 90 days were statistically significant (*p* value = 1.007*e* − 05) (Figure 2).

**Figure 3:**
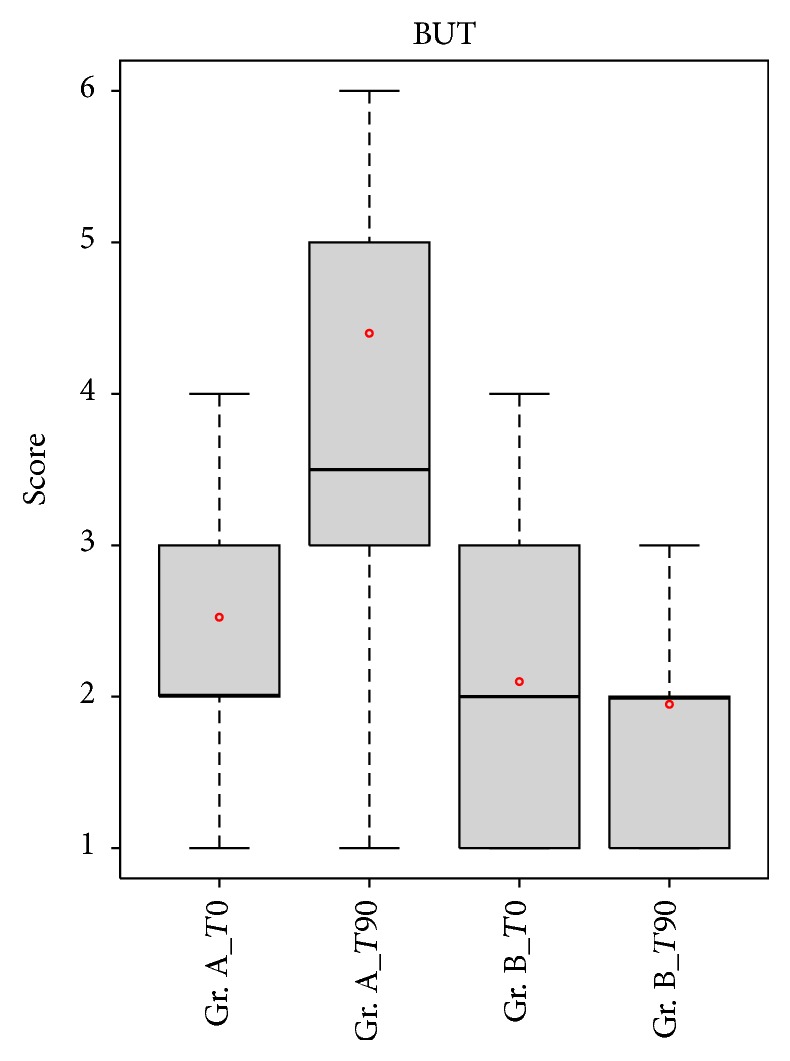
Comparison of BUT values between patients in group A (APL) and patients in group B (artificial tear). BUT increased significantly in group A patients compared with the group B patients after treatment (*p* < 0.05).

Schirmer test values did not change significantly after 90 days of treatment between the two groups (*p* value: 0.544); however, in group A, there was an increase in BUT value after treatment, compared to group B (*p* value < 0.001) (Figure 3).

**Figure 4:**
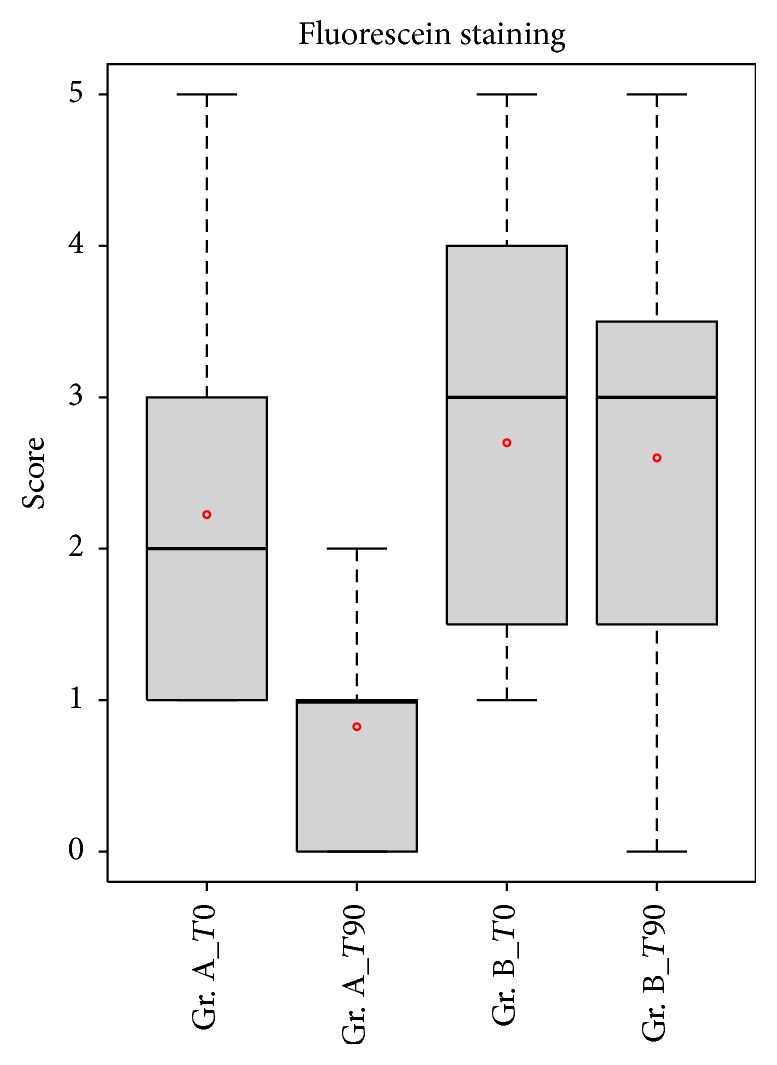
Comparison of fluorescein score between patients in group A (APL) and patients in group B (artificial tear). Fluorescein score was significantly lower in group A patients compared with the group B patients after treatment (*p* < 0.05).

Concerning the fluorescein score, the differences in the mean ocular surface staining scores between the two groups after treatment were statistically significant (*p* < 0.05). After 90 days of treatment, 80% of the examined eyes showed an Oxford score ≤1 (Figure 4).

The OPI value was significantly higher at the end of the treatment in group A compared to group B (*p* < 0.05) and a significant decrease in the mean values of posterior blepharitis (*p* < 0.05) was observed in group A compared to group B.

The BCVA significantly increased after 90 days in group A, but the improvement is not statistically significant in comparison with the treatment of group B (*p* value = 0.100).

### 3.2. In Vivo Confocal Microscopy

In vivo confocal microscopy was performed at baseline and after 90 days of treatment on 10 patients of group A (20 eyes analyzed); this subgroup (subgroup 2) was randomized from group A. Excluded patients are defined as subgroup 1.  Table 3 shows that there are not statistically significant differences between the two subgroups at baseline.

**Figure 5:**
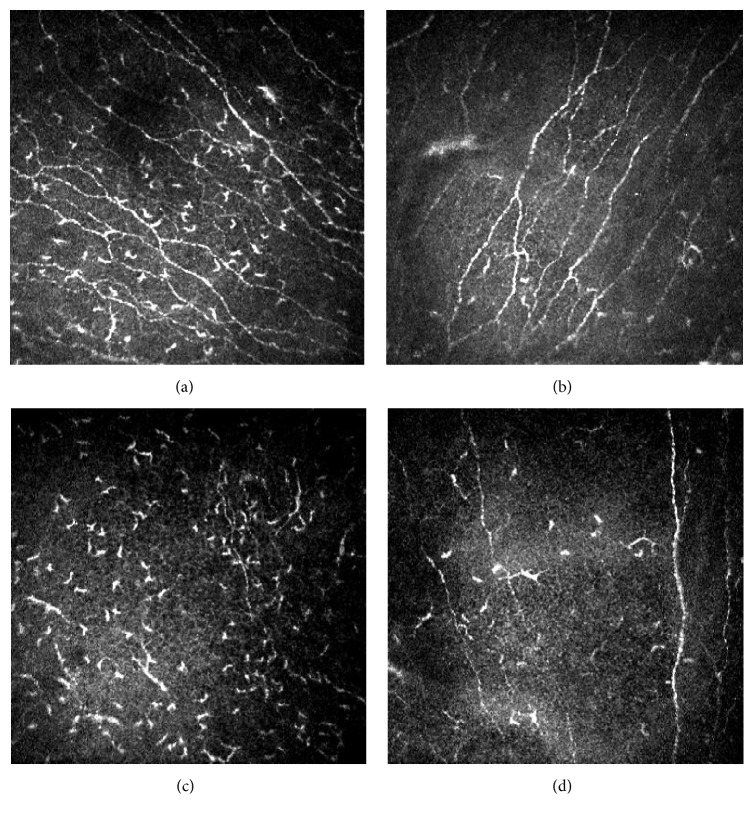
Confocal images of subbasal nerves, in central corneal sectors. At baseline (a–c), many Langerhans cells can be observed at this level; after 3-month-long treatment (b–d), a decreased number of inflammatory cells and an increased number of nerves were found. Figures show the improvement in corneal subbasal nerve plexus for two representative patients treated with APL.

After treatment, a statistically significant increase in basal epithelium cells density (*p* value = 0.005) was detected; moreover, an increase in number of subbasal nerve fibers (*p* value = 0.005) as well as an increase in the density of innervation (*p* value = 0.003) was found (Figure 5).

The assessment of gray value of the anterior stroma did not show any statistically significant changes after 90 days of treatment (*p* value = 0.222), and no differences were found in activated keratocytes density at the end of the treatment period (*p* value = 0.976).

Finally, a statistically significant decrease of Langerhans cell density (*p* value = 0.024) was detected. These results are presented in Table 4.

## 4. Discussion

Although there has been a relevant increase in knowledge regarding pathophysiology and therapeutic strategies of dry eye, severe dry eye constitutes a major management challenge. In fact, there is a large cohort of severe dry eye patients having persistent signs and symptoms despite maximal conventional therapies.

The critical role of inflammation in the pathogenesis of dry eye has suggested treatment options other than artificial tears. Topical administration of corticosteroids has been reported to improve signs and symptoms in patients with KCS [26], but these treatments cannot be used as long-term therapy and need careful monitoring, due to steroid-related complications (increased intraocular pressure and cataract). Clinical trials have shown topical cyclosporine to be effective in patients with moderate to severe dry eye, because of its ability to decrease inflammation and increase goblet cells density [27–29]. However, topical cyclosporine is correlated with several side effects, such as intolerable irritation, limiting its use [30].

It has been demonstrated that a reduction in epitheliotropic factors can lead to a loss of epithelial integrity and a delay in healing process; since standard therapy for DES such as artificial tears, corticosteroid, and cyclosporine is not able to restore the natural composition in GF of human tears, the use of hemoderivatives has gained popularity.

The autologous serum therapy is worldwide accepted [31–33], but the practicalities and published evidence were recently reviewed with the evidence that the use of AS was not associated with effects based on objective measurements. This result could be due to the presence of a high level of proinflammatory cytokine in the serum [34].

Based on the concept that platelet *α* granules are a major source of GFs, the research interest has moved to platelet-enriched plasma [9, 35]. Vitamin A, Endothelial Growth Factor (EGF), fibronectin, TGF-*β*, and nerve growth factor (NGF) are necessary for maintaining the integrity of conjunctival and corneal integrity and it has been demonstrated that LPA is rich in these GFs. A critical role seems to be played by TGF-*β*, in relation to the balance T regulatory (Treg)/Thelper 17 (Th17). TGF-*β* is a pleiotropic cytokine that can have pro- or anti-inflammatory effects depending on the context. It has been demonstrated that the correct Treg/Th17 ratio normally contributes to the maintenance of immune tolerance. Inflammatory interleukins, such as IL-6 or IL-27, can alter the balance in favor of Th17, promoting the production of interleukin-17 (IL-17) [36]. TGF-*β*, in appropriate concentrations, inhibits the priming of Th17, converting the naive T cell in Treg suppressor [37, 38].

Uncontrolled clinical studies have provided evidence of symptoms decrease and objective parameters improvement, such as impression cytology and BUT, after PRP and APL treatment in different pathologies and with a different posology or timing [10–12].

To our knowledge, this is the first randomized prospective study evaluating the effectiveness of APL. We used the standardized OSDI survey and objective clinical measures to evaluate its efficacy. Moreover, for the first time, we assessed the corneal morphological modifications after APL treatment by IVCM.

In our study, subjects without a satisfactory control of the disease with conventional topical therapy achieved improvement in symptoms after APL treatment. This improvement was significantly higher compared to patients treated with tear substitutes.

FBUT average and, in consequence, the mean value of OPI increased in patients treated with LPA, indicating greater stability and an improvement in quality of the tear film. The corneal staining score improved significantly and after 90 days of treatment 80% of patients showed an Oxford score ≤1.

BCVA did not increase significantly compared with group B, but an improvement was found in group A from the baseline. It must be considered that the decrease in visual acuity in these patients is probably due to other causes (conditioning regimen, topical and systemic steroids, and cataract) than tear film instability and therefore not reversible by platelet lysate. It has been demonstrated that the visual acuity measured by Snellen charts is not a good indicator of visual disturbances in patients suffering from dry eye. The Hartmann-Shack aberrometry and the double-pass aberrometry with OQAS (Optical Quality Analysis System) were found to be much more sensitive in quantifying visual disturbances in these patients [39].

Regarding side effects, there was only one reported conjunctivitis case during the treatment with LPA; the patient was temporarily excluded and resumed in group A after appropriate antibiotic therapy. The APL has bacteriostatic factors (*β*-lysine, lactoferrin, antibodies IgG and IgA, and lysozyme), which make superfluous addition of preservatives; however, all preparations underwent a bacterial culture before delivering.

Previous IVCM studies found morphological abnormalities in SS corneas. They described abnormal epithelial and stromal cells, decreased corneal thickness, and alterations of nerves number, density, and tortuosity as well as increased density of LCs [18, 19, 40–42].

After 3 months of treatment with APL, an increase in cell density of basal layer was found; such finding may be due to the migration of stem cells in division [43], attempting to reepithelize corneal central sectors. We observed complete resolution of corneal epithelial defect in 40% of treated eyes and an improvement in 90% of eyes.

The neuronal morphometric analysis of images captured with IVCM and processed with NeuronJ showed an increased number and density of subbasal nerves after 3 months. This improvement could be due to the direct or indirect action of nerve growth factor (NGF), present in APL. NGF in a neurotrophin has a role in nerve sprouting and damaged neurons restoring. Even if its use has shown encouraging results in few works that state its efficacy in neurite sprouting by neural cells [44–46], the chance that the APL could determine the regeneration of corneal nerves is currently discussed in literature [47]. A number of authors have recently observed that autologous serum is effective in restoring nerve topography through nerve regeneration in patients with corneal neuropathy [21, 22]. Our study showed similar results, but the limited sample and the not comparative nature of the evaluation do not allow more in-depth analyses on factors favoring this response. However, the leading mechanism for the actual nerve regeneration remains to be determined. It is possible that an improvement in epithelial healing, inflammatory response, and tear film changes could have a main role.

It is widely known that Langerhans cells migration is influenced by cytokines and chemokines [48] and that a significant increase of LCs density has been observed in the subbasal nerve plexus in patients with SS [41]. In group A patients, the density of Langerhans cells in central areas of the cornea underwent a significant reduction, suggesting the anti-inflammatory activity of platelet lysate. In support of this evidence, it has been shown that TGF-*β*, in suitable concentrations, can suppress resident DC maturation [49].

Activated stromal keratocytes density and anterior stroma gray values (Optical Units), which correspond to stromal reflectivity, did not change significantly after the treatment; this finding is probably due to the bioavailability of platelet lysate at this level, which is not known, but it could be reasonably expected to be very low (below 5%).

The main limitation of this study was the absence of evaluation of repeatability for confocal microscopy parameters. In literature, an inter- and intraobserver variance inferior to 5% [25, 50] is reported. Based on this data showing a high repeatability of IVCM, we decided not to undergo this type of analysis in our study.

The quantity of blood extraction required for the APL preparation is clearly higher than the one for AS preparation [51], in order to obtain the desired platelets concentration. However, the blood sample collection was conducted in safe conditions at the Transfusional Center of the Città della Salute e della Scienza by skilled nurses and Hb values were strictly evaluated before and after the procedure to exclude the potential risk of anemia. The blood sample was drawn once at the beginning of the study to increase patient's comfort and compliance. The optimal concentration of growth factors in the APL has not been established yet. The high interpatient variability of growth factor can potentially be responsible for differences in the efficacy of this treatment [12]. However, the preparation of any blood derivate is not standardized and the concentration of growth factors in any blood derivate is not completely clear. As a matter of fact, even the AS has been used in different studies with concentrations ranging from 20 to 100%. The minimal effective concentration of autologous hemoderivatives is still unknown and further studies should be addressed to define this value.

## 5. Conclusions

In conclusion, this study supports the hypothesis that autologous platelet lysate eye drops are effective on both subjective symptoms and objective findings, in the treatment of significant dry eye, in patients with primary Sjögren syndrome. The IVCM images added objectiveness to the evaluation suggesting that APL could be effective in restoring corneal damage by promoting epithelial and nerve regeneration and decreasing Langerhans cells. Our results encourage the use of morphological evaluations as a useful tool in the diagnosis and management of dry eye.

## Figures and Tables

**Table 1:** Patients characteristics at baseline. Group A = patients assigned to APL; group B = patients assigned to artificial tears.

	Group A	Group B	*p* value
Female, *n* (%)	19.0 (95)	10.0 (100.0)	1.00
Age, mean (SD) years	60.4 (11.68)	59.5 (13.34)	0.758
Dry eye, mean (SD) years	7.6 (6.5)	7.5 (6.2)	0.767

**Table 2:** Ophthalmological assessment.

		Group A	Group B	*p* value Gr. A versus Gr. B
OSDI				
*T*0	Media (SD)	60.56 (17.69)	60.67 (16.19)	0.821
*T*90	Media (SD)	21.75 (9.86)	53.60 (14.91)	<0.001^*∗*^
*p* value		0.000438^*∗*^	0.059336	

Fluorescein score (0–5)				
*T*0	Median (IQR)	2 (2)	3 (2.5)	0.17
*T*90	Median (IQR)	1 (2)	3 (2)	<0.001^*∗*^
*p* value		<0.001^*∗*^	0.463	

FBUT (sec)				
*T*0	Median (IQR)	2 (1)	2 (2)	0.18
*T*90	Median (IQR)	3.5 (2)	2 (1)	<0.001^*∗*^
*p* value		0.005^*∗*^	0.463	

BCVA				
*T*0	Median (IQR)	8 (4)	6 (3.5)	0.218
*T*90	Median (IQR)	8 (3.5)	6 (3.5)	0.100
*p* value		0.008^*∗*^	0.317	

Schirmer test (mm)				
*T*0	Median (IQR)	3 (2)	3 (2)	0.989
*T*90	Median (IQR)	3 (1)	3 (0.5)	0.544
*p* value		0.0611	0.328065	

Anterior blepharitis (grades 0–4)				
*T*0	Median (IQR)	1 (1)	2 (1.5)	0.092
*T*90	Median (IQR)	1 (0.5)	2 (1.5)	0.778
*p* value		0.055	0.109	

Posterior blepharitis (grades 0–4)				
*T*0	Median (IQR)	1 (1)	1.5 (1)	0.748
*T*90	Median (IQR)	1 (1)	1.5 (1.5)	0.036^*∗*^
*p* value		0.011^*∗*^	0.310	

Lipcof (grades 0–3)				
*T*0	Median (IQR)	3 (2)	3 (1)	0.419
*T*90	Median (IQR)	3 (2)	3 (1)	0.994
*p* value		1.000	1.000	

OPI (>/<1)				
*T*0	Median (IQR)	0.29	0.23	0.778
*T*90	Median (IQR)	0.53	0.25	<0.001
*p* value		<0.001^*∗*^	0.245	

*T*0 = time 0; *T*90 = time 90 days; SD = standard deviation; IQR = interquartile range; OSDI = ocular surface disease index; OPI = ocular protection index; FBUT = breakup time; BCVA = best corrected visual acuity.

^*∗*^Statistically significant results.

**Table 3:** Basal characteristics of subgroup 1 (nonevaluated by IVCM) and subgroup 2 (evaluated by IVCM).

		Subgroup 1	Subgroup 2	*p* value
Age	Media (SD)	63.67 (8.73)	57.6 (11.76)	0.692
Sex	Female : male (%)	9 : 10 (90)	10 : 0 (100)	1.000
OSDI	Media (SD)	63.73 (21.74)	56.07 (19.3)	0.397
Fluorescein score	Median (IQR)	2 (1)	1.5 (1.5)	0.744
BUT	Median (IQR)	2 (1.5)	3 (1)	0.287
Schirmer test	Median (IQR)	3 (1)	4 (1)	0.055

**Table 4:** Confocal microscopy assessments, subgroup 2.

	*T*0	*T*90	*p* value
Basal epithelial cell density (cell/mm^2^)			
Media	5810.52	6680.16	0.005^*∗*^
SD	859.78	739.91	
Nerve number (*n*/frame)			
Media	5.11	7.72	0.005^*∗*^
SD	2.60	3.62	
Nerve density (*µ*m/frame)			
Median	1070.25	1655.0	0.003^*∗*^
IQR	906.46	1631.67	
Gray value anterior stroma (OU)			
Median	50.33	52.00	0.222
IQR	11	5.65	
Activated keratocyte (cell/mm^2^)			
Media	42.04	39.08	0.976
SD	20.72	20.64	
Langerhans cells (cell/mm^2^)			
Median	34.50	31.33	0.024^*∗*^
IQR	127.33	93.34	

^*∗*^Statistically significant results.

## Additional Points

*Protocol number* is 00098937, 20/9/2013.
